# Immune heterogeneity-driven therapeutic responses to platelet-rich plasma and TRPV1 antagonism in osteoarthritis

**DOI:** 10.3389/fphar.2026.1799766

**Published:** 2026-09-07

**Authors:** Pengmin Liu, Jingwei Zhu, Yang Yang, Hailong Kou, Fei Yin, Laifu Yang

**Affiliations:** 1 Department of Rehabilitation Medicine, The First Affiliated Hospital of Henan Medical University, Weihui, China; 2 Department of Pediatric Rehabilitation Department, The First Affiliated Hospital of Henan Medical University, Weihui, China; 3 Department of Pulmonary and Critical Care Medicine, The First Affiliated Hospital of Henan Medical University, Weihui, China; 4 Department of Emergency Medicine, The First Affiliated Hospital of Henan Medical University, Weihui, China; 5 Department of Critical Care Medicine, The First Affiliated Hospital of Henan Medical University, Weihui, China

**Keywords:** immune heterogeneity, neuroimmune crosstalk, osteoarthritis, peripheral and central sensitization, platelet-rich plasma, therapeutic response, TRPV1 antagonism

## Abstract

**Introduction:**

Osteoarthritis (OA) exhibits substantial heterogeneity in synovial immune profiles, which may underlie variable analgesic responses to standard biological and neuromodulatory treatments. Remodeling the immune microenvironment while targeting nociceptive pathways may improve therapeutic consistency.

**Methods:**

Analgesic efficacy of platelet-rich plasma (PRP) combined with transient receptor potential vanilloid 1 (TRPV1) antagonism was evaluated in a monosodium iodoacetate (MIA)-induced rat OA model. Synovial immune polarization, inflammatory signaling, peripheral and central sensitization were assessed mechanistically. Clinical outcomes were analyzed in a cohort of 140 OA patients receiving combination or single-agent therapy.

**Results:**

Combined PRP and TRPV1 blockade produced stronger analgesia than either treatment alone in rats. The combination shifted synovial macrophages toward an anti-inflammatory M2 phenotype, suppressed NF-κB signaling, and reduced pro-inflammatory cytokines. It also decreased dorsal root ganglion excitability and spinal glial activation, indicating reduced peripheral and central sensitization. In OA patients, combination therapy yielded superior pain relief, functional improvement, and reduced sensitization-related phenotypes.

**Discussion:**

These findings support a neuroimmune model in which synovial immune heterogeneity influences treatment responsiveness. Concurrent immune remodeling via PRP and nociceptor inhibition via TRPV1 antagonism represents a promising strategy to achieve more reliable analgesia in OA.

## 1 Introduction

Osteoarthritis (OA) is a highly prevalent degenerative joint disease and a leading contributor to disability worldwide. Increasing evidence indicates that OA is not only a mechanical disorder driven by cartilage wear but a whole-joint disease characterized by chronic low-grade inflammation and reciprocal interactions among the immune, neural, and structural compartments. Clinically, pain severity often shows a weak correspondence with radiographic changes, indicating that structural degeneration alone does not account for symptom burden or treatment responsiveness. This mismatch points to biological heterogeneity within the joint microenvironment as a plausible driver of divergent pain phenotypes and variable therapeutic outcomes. Current OA management still relies heavily on nonsteroidal anti-inflammatory drugs (NSAIDs), which provide incomplete symptomatic relief, do not modify disease progression, and may increase gastrointestinal and cardiovascular risks when used for a long term (Zhu et al., 2024; Li et al., 2024; Mobasheri et al., 2024).

OA pain is increasingly conceptualized as a neuroimmune process sustained by heterogeneous inflammatory states in the synovium. In early disease, interindividual differences in synovial cytokines and chemokines can shape the local immune milieu, lower nociceptor activation thresholds in synovial and subchondral tissues, and promote peripheral sensitization (Song et al., 2025). Transient receptor potential vanilloid 1 (TRPV1), a nonselective cation channel expressed on primary sensory neurons, integrates thermal, acidic, and inflammatory cues and represents a key node linking immune signaling to nociceptor excitability (Yoshioka et al., 2024).

Persistent peripheral nociceptive input can further cause maladaptive plasticity in the spinal dorsal horn. With sustained afferent drive, microglial and astrocytic activation may increase, facilitating central sensitization and amplifying pain transmission (Du and Liang, 2025). Importantly, central sensitization varies across patients even when structural damage appears comparable, thus reinforcing the view that neuroimmune heterogeneity contributes to pain variability and inconsistent responses to treatment. Accordingly, approaches that address both immune-driven peripheral sensitization and central amplification mechanisms are increasingly being considered necessary for more durable analgesia in OA (Wu WS. et al., 2025).

Platelet-rich plasma (PRP) is an autologous biologic containing multiple growth factors (e.g., transforming growth factor-β, platelet-derived growth factor, and insulin-like growth factor) and has been widely explored for supporting cartilage homeostasis and joint repair. Experimental and clinical evidence indicates that PRP can protect chondrocytes, promote extracellular matrix synthesis, and suppress catabolic pathways by rebalancing the joint microenvironment (Sánchez et al., 2025). Beyond the potential structural effects, PRP may also influence immune cells, including macrophages, and promote anti-inflammatory polarization, thereby offering a route to remodel synovial immune heterogeneity.

However, analgesic responses to PRP are inconsistent. Some patients show delayed or inadequate pain relief, whereas others experience transient symptom exacerbation early after treatment (Ro et al., 2024). This variability cannot be explained solely by procedural factors and may reflect baseline differences in synovial immune states. In contrast, TRPV1 antagonism directly targets neural sensitization by suppressing nociceptive signal transduction and neurogenic inflammation, which may induce faster analgesia. Yet, TRPV1 blockade alone is not expected to correct the underlying inflammatory microenvironment. These complementary mechanisms raise the possibility that combining PRP-mediated immune remodeling with TRPV1 antagonism could reduce response heterogeneity by simultaneously targeting the immune and neural drivers of OA pain (Cai et al., 2025; Kotlier et al., 2024).

We here investigated whether immune heterogeneity in OA influences therapeutic sensitivity to PRP and TRPV1 antagonism and whether combination therapy produces superior outcomes. Using a monosodium iodoacetate (MIA)-induced rat OA model, we assessed synovial immune remodeling (macrophage polarization and inflammatory signaling), peripheral sensitization (dorsal root ganglion excitability), and central sensitization (spinal dorsal horn glial activation) under monotherapy *versus* combination therapy. We further extended these mechanistic observations to a clinical cohort to evaluate whether the combined approach is associated with improved pain/function outcomes and reduced sensitization-related phenotypes compared with single-agent treatment.

## 2 Methods

### 2.1 Study design and workflow

We adopted a translational sequential design to examine whether synovial immune heterogeneity contributes to variable analgesic responses in OA and whether combining PRP with TRPV1 antagonism can concurrently remodel the synovial immune microenvironment and attenuate peripheral-to-central sensitization. The study comprised two linked components: (i) a mechanistic animal experiment designed to establish a closed-loop evidence chain across the immune, neural, and structural levels and (ii) a real-world clinical cohort designed to test translational consistency in pain/function outcomes and sensitization-related phenotypes.

In this animal study, an MIA-induced rat OA model was used to generate stable pain hypersensitivity and cartilage degeneration. The animals received PRP, a TRPV1 antagonist, the combination therapy, or appropriate controls. Longitudinal behavioral measures captured the time course of mechanical and thermal hypersensitivity and functional impairment. Synovial immunophenotyping and inflammatory signaling assays were used to quantify immune-state remodeling, with a particular focus on macrophage polarization. Peripheral sensitization was evaluated along the joint–dorsal root ganglion (DRG) axis, and central sensitization was assessed using spinal dorsal horn neuronal and glial activation markers. Structural outcomes were quantified using histological staining and cartilage matrix markers to distinguish analgesic effects from tissue protection.

**FIGURE 1:**
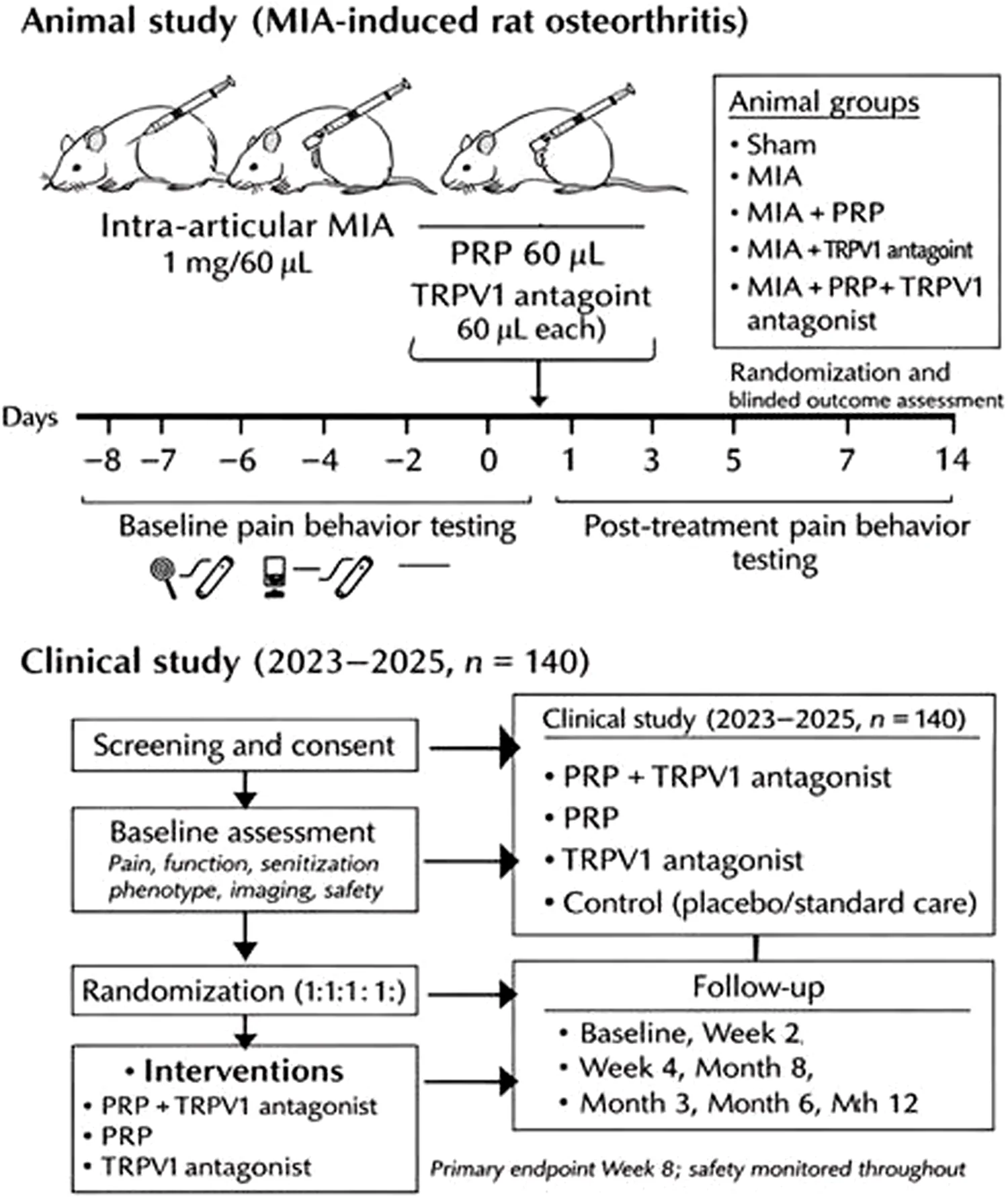
Timeline of the sequential animal-to-clinical study of PRP + TRPV1 antagonism in osteoarthritis. Top (animal study): after a 7-day acclimatization period, MIA was injected intra-articularly on day 0. Interventions were initiated on day 7 (PRP, TRPV1 antagonist, or the combination). Behavioral assessments were performed at days −1, 3, 7, 10, 14, 18, and 21. Synovial immune and cytokine readouts were assessed at day 5 and day 21, and DRG (L3–L5) and spinal dorsal horn tissues (L4–L5) were collected at day 21. Bottom (clinical cohort): a real-world, prospective observational cohort was enrolled between 2023 and 2025 (n = 140). Participants received PRP alone, TRPV1 antagonism alone, or the combined regimen under routine clinical care. Outcomes were assessed at week 2, week 6, and week 12 (±7 days), including pain/function (VAS and WOMAC) and sensitization phenotypes (PPT, CSI-25, and CPM).

In the clinical cohort (2023–2025), OA patients were followed-up with prespecified endpoints for pain and function, alongside sensitization-related phenotypes intended to map onto the animal mechanistic readouts. Repeated-measures models were used to estimate time-varying treatment effects and test whether the combination therapy produced benefits beyond additivity after adjusting for baseline covariates and potential confounders. The overall workflow and schedule are summarized in Figure 1.

### 2.2 Materials

To ensure reproducibility and comply with reporting requirements, the key reagents, antibodies, assay kits, and core equipment are specified here with the manufacturer and catalog numbers when applicable. MIA used for OA induction was purchased from Sigma-Aldrich/MilliporeSigma (Cat. No. I2512). The selective competitive TRPV1 antagonist SB-366791 was obtained from Tocris Bioscience (Cat. No. 1615) and prepared in the vehicle described in the *Methods* section. Autologous PRP was prepared in-house from whole blood using a standardized two-step centrifugation protocol; because it was not a commercial product, a catalog number is not applicable, and reproducibility is ensured through explicit reporting of the preparation parameters and administration schedule.

ELISA kits were used for synovial cytokine quantification of TNF-α (R&D Systems/Bio-Techne, RTA00), IL-1β (R&D Systems/Bio-Techne, RLB00), IL-6 (R&D Systems/Bio-Techne, R6000B), and IL-10 (R&D Systems/Bio-Techne, R1000) following the manufacturers’ instructions. For immunophenotyping and mechanistic readouts, primary antibodies included iNOS (Abcam, ab15323), CD86 (Proteintech, 30691-1-AP), CD206 (Proteintech, 18704-1-AP), and Arg1 (Proteintech, 16001-1-AP) for macrophage polarization; TRPV1 (Alomone Labs, ACC-030), NaV1.8 (Alomone Labs, ASC-016), CGRP (Abcam, ab36001), phospho-ERK1/2 (Cell Signaling Technology, 4370), and total ERK1/2 (Cell Signaling Technology, 4695) for DRG sensitization signaling; and c-Fos (Cell Signaling Technology, 2250), Iba1 (FUJIFILM Wako, 019-19741), and GFAP (Dako/Agilent, Z0334) for dorsal horn neuronal and glial activation. The cartilage matrix and degeneration markers were assessed using antibodies against collagen II (Abcam, ab34712), aggrecan (Abcam, ab3778), and MMP-13 (Abcam, ab39012).

For protein assays and immunoblotting, the total protein concentration was determined using a BCA protein assay kit (Thermo Fisher Scientific, 23225), and PVDF membranes (Millipore Immobilon-P, 0.45 μm, IPVH00010) were used for immunoblot transfer, unless otherwise specified. For histological evaluation, Safranin O (Sigma-Aldrich, S8884) and Fast Green FCF (Sigma-Aldrich, F7252) were used for Safranin O/Fast Green staining, respectively. For behavioral testing and sensitization phenotyping, mechanical hypersensitivity was assessed using an electronic von Frey aesthesiometer (IITC Life Science, 2390 series, or equivalent). In the clinical cohort, pressure pain threshold (PPT) testing was performed using a handheld digital pressure algometer with a 1-cm^2^ probe (Somedic SenseLab, Algometer, or equivalent), and for conditioned pain modulation (CPM), we used a cold-pressor conditioning stimulus delivered by circulating cold water maintained at 10 °C using a circulating water bath (e.g., JULABO PURA series or equivalent).

### 2.3 Animal model, interventions, and experimental groups

A rat knee OA model was induced using intra-articular MIA, which is a well-established approach that produces reproducible cartilage degeneration and sensitization-associated pain behaviors within a relatively short timeframe (Guzman et al., 2003). Adult male Sprague–Dawley rats (8 weeks old; 220 g–250 g) were acclimatized for 7 days prior to baseline testing. OA induction was performed *via* aseptic intra-articular injection of MIA (Sigma-Aldrich/MilliporeSigma; Cat. No. I2512) (2 mg in 50 μL sterile saline) into the right knee, while sham controls received 50 μL sterile saline.

After induction, the animals were randomly allocated into five groups using computer-generated block randomization, namely, the sham control, OA model control, PRP monotherapy, TRPV1 antagonist monotherapy, and combined PRP + TRPV1 antagonist treatment groups (n = 10 per group). Investigators performing behavioral testing, tissue processing, and quantitative analyses were blinded to group allocation.

PRP preparation and administration: PRP was prepared using a standardized two-step centrifugation protocol to obtain leukocyte-poor PRP with a target platelet enrichment of approximately 4–5× above baseline and controlled leukocyte content, thereby reducing preparation-related variability (Filardo et al., 2018). PRP was gently resuspended and used fresh within a predefined time window. PRP (100 μL) was administered *via* intra-articular injection into the OA knee at day 7 and day 14 post-induction. Control injections consisted of volume-matched sterile saline administered on the same schedule.

TRPV1 antagonism: To ensure reproducibility of TRPV1 blockade, SB-366791 (Tocris Bioscience; Cat. No. 1615), a selective competitive TRPV1 antagonist, was used. SB-366791 was dissolved in a vehicle consisting of 10% DMSO, 40% PEG300, 5% Tween-80, and 45% saline and administered intraperitoneally at 1.0 mg kg^-1^ once daily from day 7 to day 21 post-induction. Vehicle-matched injections were administered to the corresponding control groups.

Animals were monitored throughout the study for body weight, grooming/activity, and local joint reactions. Prespecified exclusion criteria included overt joint infection, severe procedure-related complications, or inability to complete behavioral testing; exclusions were documented before unblinding.

### 2.4 Animal outcome assessments across immune–neural–structural levels

Animal outcomes were selected to capture the treatment effects across immune remodeling, peripheral sensitization, central sensitization, and structural degeneration, with emphasis on cross-level coupling between the synovial immune states and the peripheral-to-central sensitization axis.

#### 2.4.1 Behavioral and functional assessments

Mechanical hypersensitivity and thermal hypersensitivity were assessed longitudinally using standardized withdrawal measures. Testing was performed at baseline (day −1) and post-induction on day 3, day 7, day 10, day 14, day 18, and day 21. Weight-bearing distribution and gait-related measures were collected at matched time points to quantify pain-related unloading and functional impairment. Mechanical hypersensitivity was assessed using an electronic von Frey aesthesiometer (IITC Life Science, 2390 series or equivalent), and thermal hypersensitivity was assessed using standardized withdrawal paradigms implemented under a prespecified testing SOP.

#### 2.4.2 Synovial immune microenvironment and inflammatory signaling

Synovial tissue was analyzed to quantify macrophage polarization and inflammatory signaling (Zhang et al., 2018). M1-like macrophages were indexed using iNOS (Abcam; ab15323) and CD86 (Proteintech; 30691-1-AP), and M2-like macrophages were indexed using CD206 (Proteintech; 18704-1-AP) and Arg1 (Proteintech; 16001-1-AP). Quantification was performed by immunofluorescence or immunohistochemical analyses with standardized acquisition settings, predefined thresholding, and blinded scoring. Synovial cytokines reflecting pro- and anti-inflammatory states (TNF-α, IL-1β, IL-6, and IL-10) were quantified using ELISA or equivalent immunoassays (R&D Systems/Bio-Techne: TNF-α RTA00, IL-1β RLB00, IL-6 R6000B, and IL-10 R1000).

To quantify pathway-level immune remodeling, key inflammatory signaling readouts were assessed in synovial tissue using protein assays and/or immunostaining focusing on NF-κB pathway activation (e.g., p-p65/p65 ratio) and the related downstream mediators. All quantitative analyses were performed blinded to group identity.

#### 2.4.3 Peripheral sensitization along the joint–DRG axis

Ipsilateral lumbar DRGs (L3–L5) were harvested at day 21 to quantify sensitization-related markers (Fernandes et al., 2016). Protein expressions of TRPV1 (Alomone Labs; ACC-030), Nav1.8 (Alomone Labs; ASC-016), CGRP (Abcam; ab36001), and pERK/ERK (Cell Signaling Technology: pERK1/2 4370; total ERK1/2 4695) were measured by Western blotting and/or immunostaining. The total protein concentration was determined using a BCA protein assay kit (Thermo Fisher Scientific; 23225), and PVDF membranes (Millipore Immobilon-P, 0.45 μm, IPVH00010) were used for immunoblot transfer unless otherwise specified. Analyses were normalized to appropriate housekeeping controls, and quantification was performed under blinded conditions.

#### 2.4.4 Central sensitization in the spinal dorsal horn

Ipsilateral lumbar dorsal horn tissue (L4–L5) was harvested at day 21 to assess neuronal activation and glial reactivity (Cao and Zhang, 2008). Neuronal excitation was indexed using c-Fos (Cell Signaling Technology; 2250), microglial activation was indexed using Iba1 (FUJIFILM Wako; 019-19741), and astrocytic activation was measured using GFAP (Dako/Agilent; Z0334). Markers were quantified by immunostaining and/or protein assays with blinded analysis. Central readouts were interpreted together with synovial cytokine profiles and DRG sensitization markers to assess coordinated attenuation of the peripheral and central sensitization signatures under combination therapy.

#### 2.4.5 Structural outcomes

Knee joints were processed for Safranin O/Fast Green staining using Safranin O (Sigma-Aldrich; S8884) and Fast Green FCF (Sigma-Aldrich; F7252) and evaluated using an OARSI-aligned semi-quantitative scoring approach by two blinded observers. Cartilage matrix homeostasis was additionally assessed using markers including collagen II (Abcam; ab34712), aggrecan (Abcam; ab3778), and MMP-13 (Abcam; ab39012) to evaluate the anabolic–catabolic balance.

### 2.5 Clinical cohort, outcome measures, and sensitization phenotypes

#### 2.5.1 Clinical cohort and study design

A real-world, prospective observational cohort of 140 patients with knee OA was enrolled between 2023 and 2025 (Belk et al., 2021). Eligible participants met the clinical and imaging criteria for knee OA and were able to complete longitudinal assessments. The key exclusion criteria included inflammatory arthritis, recent knee surgery, active infection, intra-articular injection within the preceding 3 months, severe systemic disease precluding follow-up, or ongoing neuropathic pain disorders that could confound sensitization phenotyping.

Treatment allocation reflected routine clinical decision-making with protocolized documentation rather than randomization. Patients received one of three regimens: PRP alone, TRPV1 antagonism alone, or PRP + TRPV1 antagonism. The treatment choice was determined before baseline outcome assessment and recorded along with the clinical rationale. Because blinding of injection-based interventions is not feasible in a real-world setting, outcome assessments were standardized and performed by trained evaluators following a prespecified assessment script; data analysts were blinded to the treatment group labels during primary modeling.

#### 2.5.2 Bias control and confounding management

To reduce selection bias and confounding that are inherent to non-randomized treatment assignment, the cohort used (i) prespecified eligibility criteria and consistent follow-up windows; (ii) standardized outcome measurement protocols with assessor training; (iii) comprehensive baseline characterization to enable covariate adjustment. Baseline covariates included age, sex, body mass index (BMI), radiographic grade, symptom duration, baseline pain/function scores, prior injections, baseline analgesic use, and comorbidity burden. Rescue medication use during follow-up was recorded at each visit and incorporated as a time-varying covariate or as a sensitivity-analysis restriction. Robustness analyses were conducted using propensity-score weighting (inverse probability of treatment weighting) based on baseline covariates to balance group differences, with standardized mean differences used to evaluate the covariate balance.

#### 2.5.3 Follow-up schedule

Assessments were performed at baseline (pre-treatment) and at prespecified post-treatment time points to capture both early and sustained clinical responses. Follow-up visits were scheduled at week 2, week 6, and week 12, with an allowable visit window of ±7 days. All sensory testing was performed in a fixed sequence at each visit to reduce order effects.

#### 2.5.4 Clinical endpoints

The prespecified primary endpoint was pain improvement, which was assessed using the visual analog scale (VAS, 0–10) and the WOMAC pain subscale at each follow-up visit. The secondary clinical endpoints included functional improvement measured by WOMAC function and patient-reported global improvement. Structural or imaging outcomes, when available, were treated as exploratory endpoints.

#### 2.5.5 Sensitization phenotypes and quantitative sensory testing

To map the clinical findings onto the animal mechanistic readouts, sensitization-related phenotypes were assessed using quantitative sensory testing (QST) and validated questionnaires.

Pressure pain threshold: PPT was measured using a handheld digital pressure algometer with a 1-cm^2^ probe (Somedic SenseLab, algometer, or equivalent). Testing sites included two peri-articular knee sites (the medial joint line and patellar tendon region) and one remote control site (contralateral mid-tibialis anterior). Pressure was increased at a constant rate of 30 kPa s^-1^. The participants indicated the moment the sensation first became painful; the corresponding value (kPa) was recorded as the PPT. Three trials per site were obtained with 30-s intervals, and the mean value was used for analysis.

Central sensitization symptoms: Central sensitization symptom burden was quantified using the Central Sensitization Inventory (CSI-25; total score 0–100), with higher scores indicating greater sensitization-related symptomatology. The questionnaire was administered at baseline and at each follow-up visit under standardized instructions.

Conditioned pain modulation: CPM was assessed to estimate the descending inhibitory control. PPT at the remote control site served as the test stimulus. A cold-pressor task was used as the conditioning stimulus; the participant immersed the contralateral hand up to the wrist in circulating cold water maintained at 10 °C for 60 s, delivered using a circulating water bath (e.g., JULABO PURA series or equivalent). PPT was measured immediately before conditioning and during the final 20 s of the cold-pressor exposure. The CPM effect was defined as ΔPPT = PPT_during − PPT_pre (kPa), with positive values indicating greater inhibitory modulation. If a participant withdrew the hand early due to intolerance, the achieved conditioning duration was recorded and included as an adjustment variable in sensitivity analyses.

#### 2.5.6 Safety monitoring and rescue medication

Adverse events were recorded at each visit and *via* interim contact as needed, with particular attention provided to injection-related pain flare, swelling, local infection, and systemic symptoms. Rescue analgesics were permitted according to a prespecified plan, and any NSAID/opioid use was recorded with the dose and frequency. Rescue medication use was incorporated into longitudinal models and was additionally examined in sensitivity analyses excluding visits with protocol-deviating rescue use.

### 2.6 Statistical analysis plan

Longitudinal outcomes were analyzed using repeated-measures models to characterize heterogeneity in the treatment response over time and test whether the combination therapy trajectory differed from monotherapy trajectories. Continuous variables were summarized using appropriate distribution-aware statistics, and between-group comparisons were conducted using parametric or nonparametric methods as appropriate.

For clinical longitudinal outcomes (VAS, WOMAC pain/function, PPT, CSI, and CPM), linear mixed-effects models were fitted with random intercepts for the participants to account for within-subject correlation. Fixed effects included time, treatment, and the treatment-by-time interaction, adjusting for baseline outcome values and prespecified confounders (age, sex, BMI, radiographic grade, symptom duration, prior injections, baseline analgesic use, and rescue medication use). Robustness analyses were conducted using propensity-score weighting to evaluate the stability of the estimated treatment effects under improved covariate balance. Effect sizes with 95% confidence intervals were reported to quantify the magnitude and variability of the response.

To test whether the combined PRP + TRPV1 approach produced effects beyond additivity, the models included the main effects for PRP and TRPV1 antagonism and their interaction term (PRP × TRPV1). In addition, synergy was operationalized in an additive-improvement framework, where the expected additive improvement was defined as the sum of improvements by monotherapy relative to the OA model control, (PRP − model) + (TRPV1 − model), while the observed improvement owing to combination therapy was defined as (combination − model). A synergy index was computed as (observed − expected), and statistical evidence for non-additivity was assessed primarily by the PRP × TRPV1 interaction term.

For mechanistic endpoint families, multiplicity was controlled using the false discovery rate (Benjamini–Hochberg) correction within each endpoint family. Correlation analyses were conducted to examine the associations among synovial inflammatory markers, DRG sensitization indices, and spinal dorsal horn markers and were interpreted as cross-level coupling evidence rather than definitive causal effects.

## 3 Results

### 3.1 Pain relief in rats

In the MIA-induced OA model, rats rapidly developed stable pain-related behavioral changes, including reduced weight-bearing on the affected limb, decreased mechanical paw withdrawal threshold, and shortened thermal withdrawal latency. Compared with sham-operated rats, the MIA-treated rats exhibited persistent hypersensitivity throughout the observation period, establishing a sustained sensitization-like baseline for evaluating treatment responsiveness.

**FIGURE 2:**
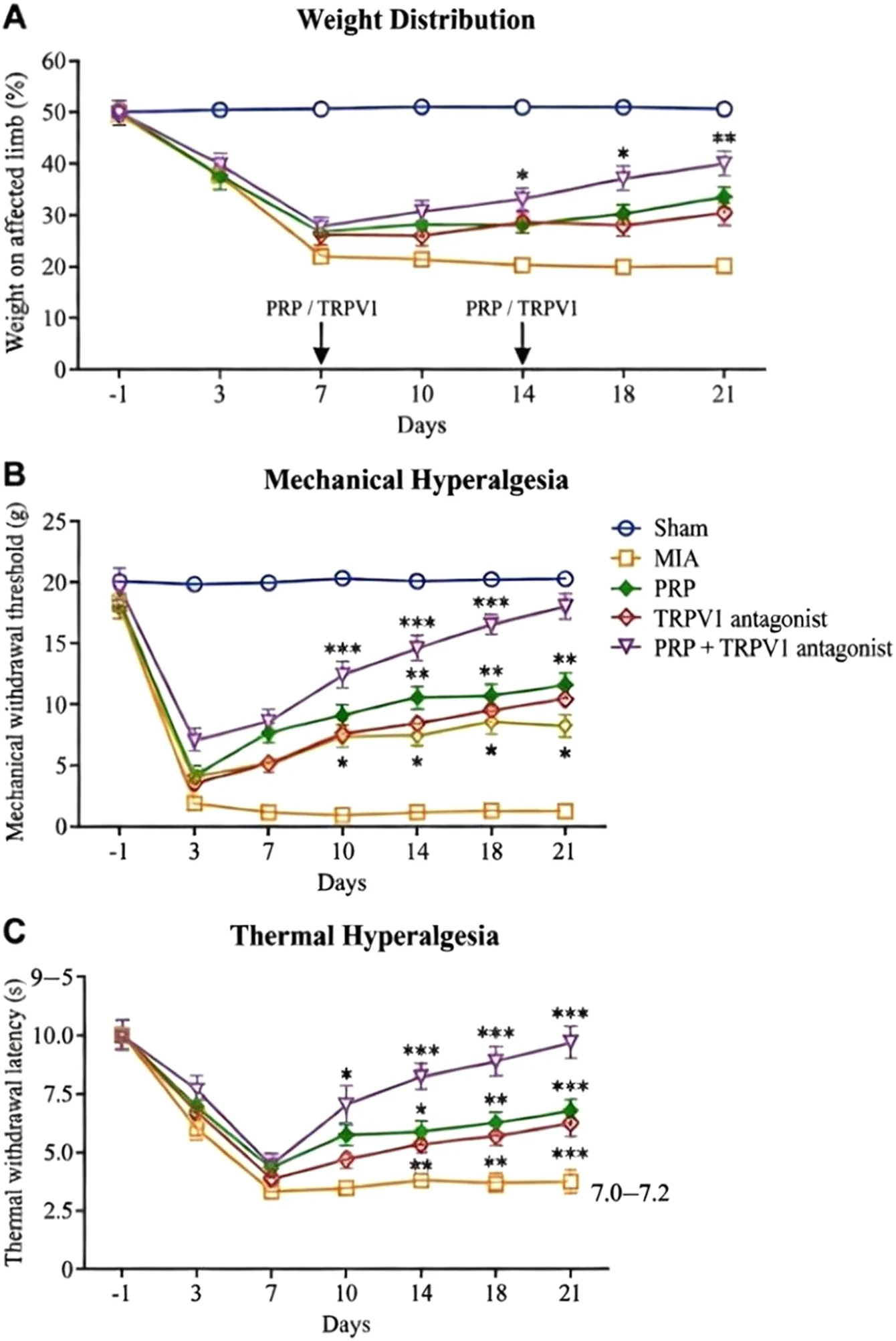
PRP + TRPV1 antagonism attenuates mechanical and thermal hypersensitivity in MIA-induced osteoarthritis model rats. Pain-related behaviors were assessed longitudinally. **(A)** Weight-bearing distribution on the affected limb (weight-bearing asymmetry). **(B)** Mechanical withdrawal threshold measured using von Frey filaments. **(C)** Thermal withdrawal latency measured using the plantar test. Data are presented as mean ± SEM (n = 10 per group). MIA, monosodium iodoacetate; PRP, platelet-rich plasma; TRPV1, transient receptor potential vanilloid 1.

In the MIA-induced OA model, behavioral assessments revealed distinct therapeutic response patterns. PRP monotherapy produced a gradual improvement in the weight-bearing distribution on the affected limb (Figure 2A), whereas the combination of PRP and TRPV1 antagonism achieved significantly greater recovery at later time points (*P < 0.05 and **P < 0.01). In the mechanical withdrawal threshold (Figure 2B), combination therapy induced the most pronounced and sustained elevation, which was markedly superior to that produced by either of the monotherapies (***P < 0.001). Similarly, thermal withdrawal latency (Figure 2C) showed the greatest enhancement under combination treatment, approaching sham levels by day 21, with high statistical significance (***P < 0.001). Overall, PRP combined with TRPV1 antagonism produced the largest and most stable improvements across all the behavioral readouts.

### 3.2 Synovial macrophage polarization

**FIGURE 3:**
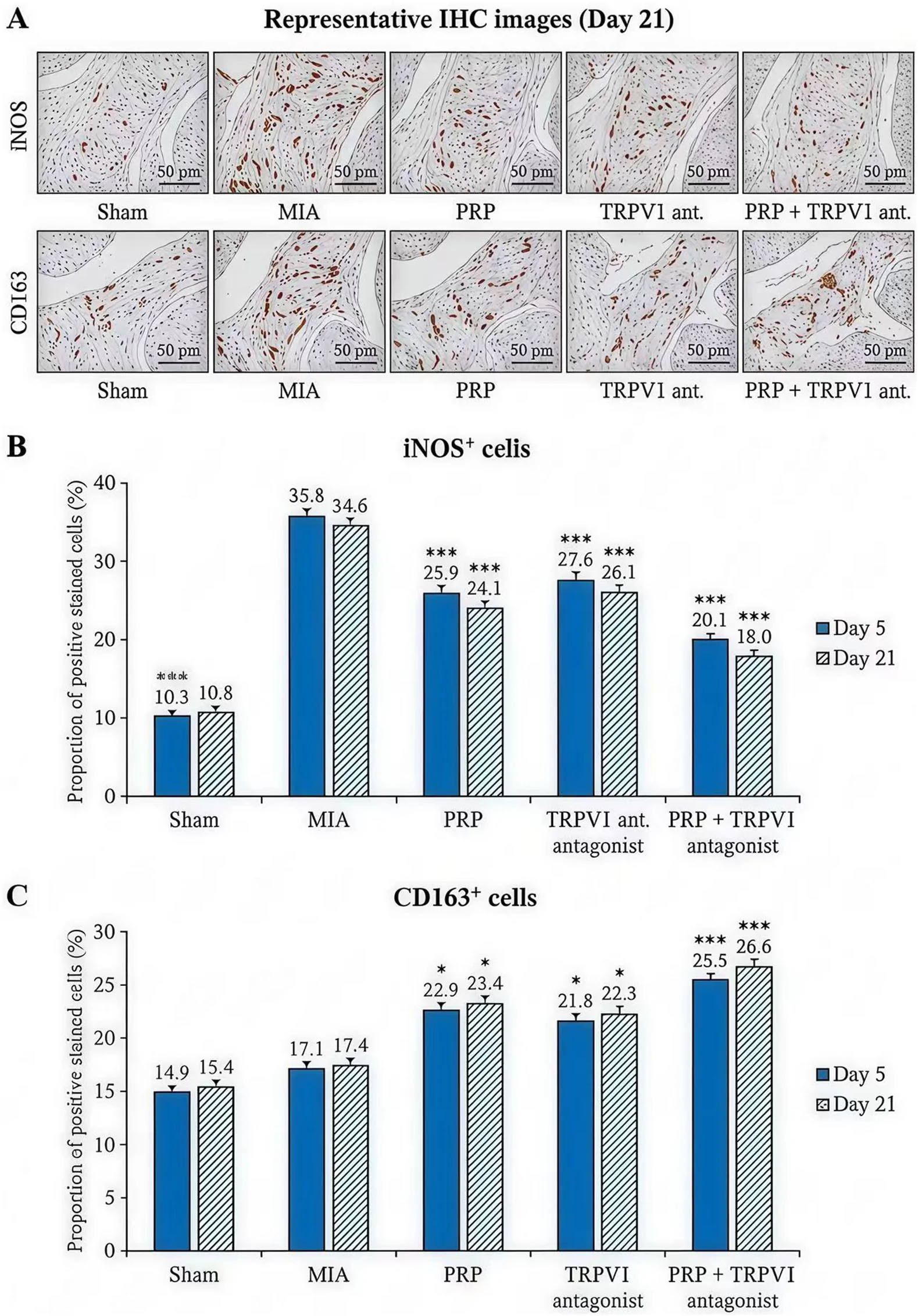
Combination therapy shifts synovial macrophage polarization toward an anti-inflammatory phenotype in MIA-induced osteoarthritis. **(A)** Representative immunohistochemistry images of synovial tissue showing iNOS (M1-associated marker) and CD163 (M2-associated marker) at day 21. Scale bar = 50 μm. **(B)** Quantification of iNOS-positive cells at day 5 and day 21. **(C)** Quantification of CD163-positive cells at day 5 and day 21. Data are presented as the mean ± SEM (n = 10 per group).

In MIA-induced OA, the synovial immune microenvironment showed a pronounced pro-inflammatory profile. Immunohistochemistry demonstrated increased iNOS-positive cells with relatively fewer CD163-positive cells in the MIA group, which is consistent with an M1-skewed polarization state (Figure 3A).

Both PRP and TRPV1 antagonist monotherapies partially shifted this profile, reducing iNOS-positive staining and increasing CD163-positive staining, although the magnitude of change varied across animals. The combination treatment produced the most consistent shift, with stronger suppression of M1-associated signals and greater enrichment of M2-associated markers than with either of the monotherapies (Figures 3B,C; Table 1).

**TABLE 1:** Synovial macrophage polarization markers (iNOS and CD163) across the experimental groups in MIA-induced osteoarthritis model rats.

Group	n	iNOS+ (%) day 5 (mean ± SEM)	*P* Vs. MIA	iNOS+ (%) day 21 (mean ± SEM)	*P* Vs. MIA	CD163+ (%) day 5 (mean ± SEM)	*P* Vs. MIA	CD163+ (%) day 21 (mean ± SEM)	P Vs. MIA
Sham	10	10.3 ± 0.7	<0.001	10.8 ± 0.6	<0.001	14.9 ± 0.8	0.190	15.4 ± 0.7	0.165
MIA	10	35.8 ± 1.1	Ref	34.6 ± 1.2	Ref	17.1 ± 0.9	Ref	17.4 ± 0.8	Ref
PRP	10	25.9 ± 1.0	0.002	24.1 ± 0.9	0.001	22.9 ± 1.0	0.003	23.4 ± 0.9	0.002
TRPV1 antagonist	10	27.6 ± 0.9	0.010	26.1 ± 1.0	0.006	21.8 ± 0.9	0.014	22.3 ± 0.8	0.010
PRP + TRPV1 antagonist	10	20.1 ± 0.8	<0.001	18.0 ± 0.7	<0.001	25.5 ± 1.0	<0.001	26.6 ± 0.9	<0.001
Overall *P* (one-way ANOVA)	​	​	<0.001	​	<0.001	​	0.005	​	0.004

Footnote: data are expressed as the mean ± SEM. *P-*values indicate *post hoc* comparisons *versus* the MIA group within the same time point. The overall *P-*values are from one-way ANOVA at each time point.

### 3.3 Synovial cytokine remodeling

**FIGURE 4:**
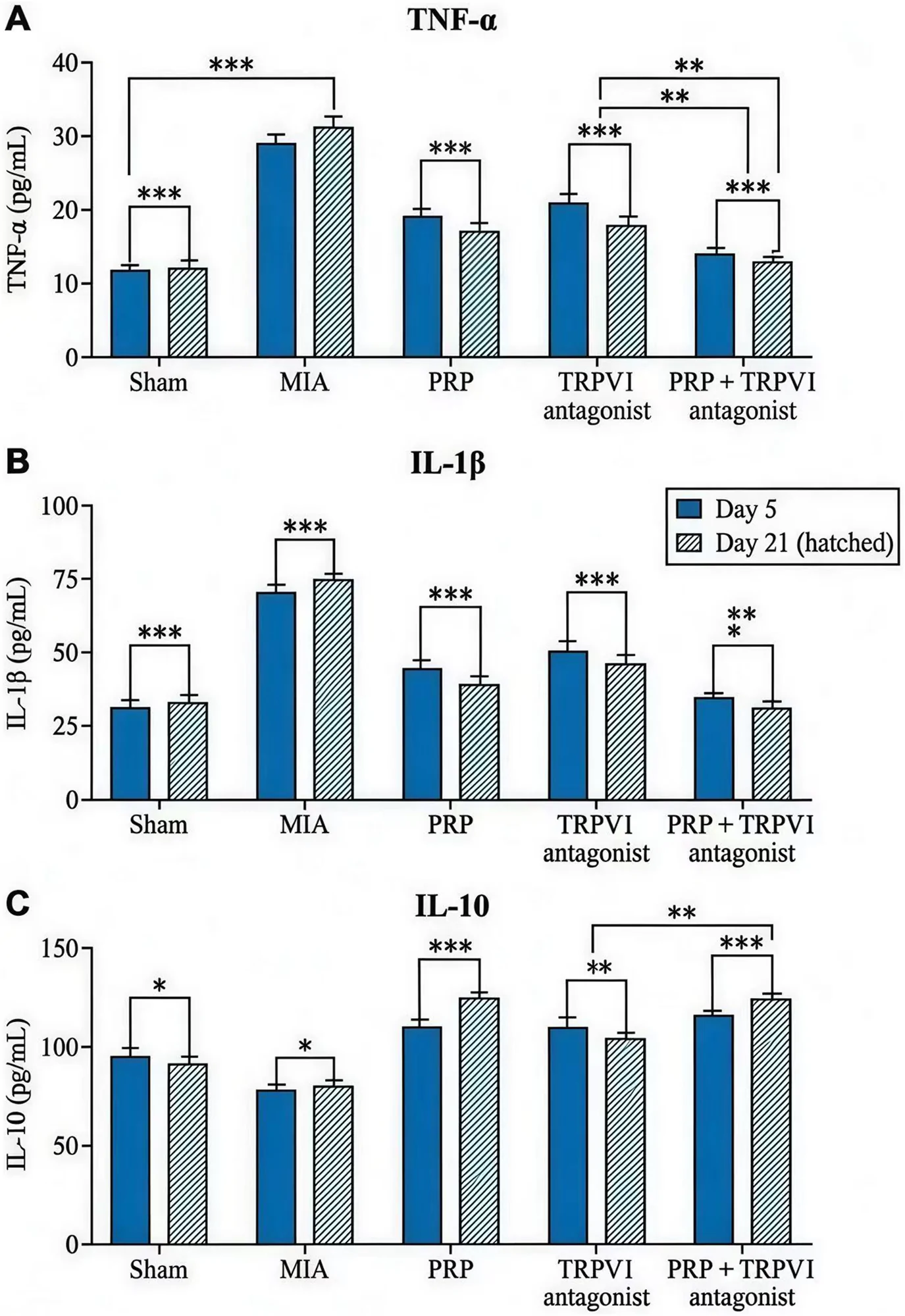
Combination therapy remodels the synovial cytokine milieu in joints with osteoarthritis. ELISA was used to quantify cytokine concentrations in joint tissues at day 5 and day 21. **(A)** TNF-α. **(B)** IL-1β. **(C)** IL-10. Data are presented as mean ± SEM (n = 10 per group).

To link the immune status to treatment response, synovial cytokines were quantified at prespecified time points. After MIA induction, TNF-α and IL-1β levels were elevated, whereas that of IL-10 remained relatively low, which is consistent with a sustained pro-inflammatory synovial milieu. PRP reduced TNF-α and IL-1β levels and increased that of IL-10, indicating partial restoration toward an anti-inflammatory balance. TRPV1 antagonism also reduced pro-inflammatory cytokines. The combination therapy produced the most stable cytokine remodeling, with stronger suppression of TNF-α and IL-1β levels and increased IL-10 compared to either of the monotherapies (Figures 4A–C).

### 3.4 NF-κB pathway activation

Canonical NF-κB pathway activation was assessed by phosphorylation of IκBα and NF-κB p65. After MIA induction, p-IκBα and p-p65 levels increased, which is consistent with sustained inflammatory pathway activation.

**FIGURE 5:**
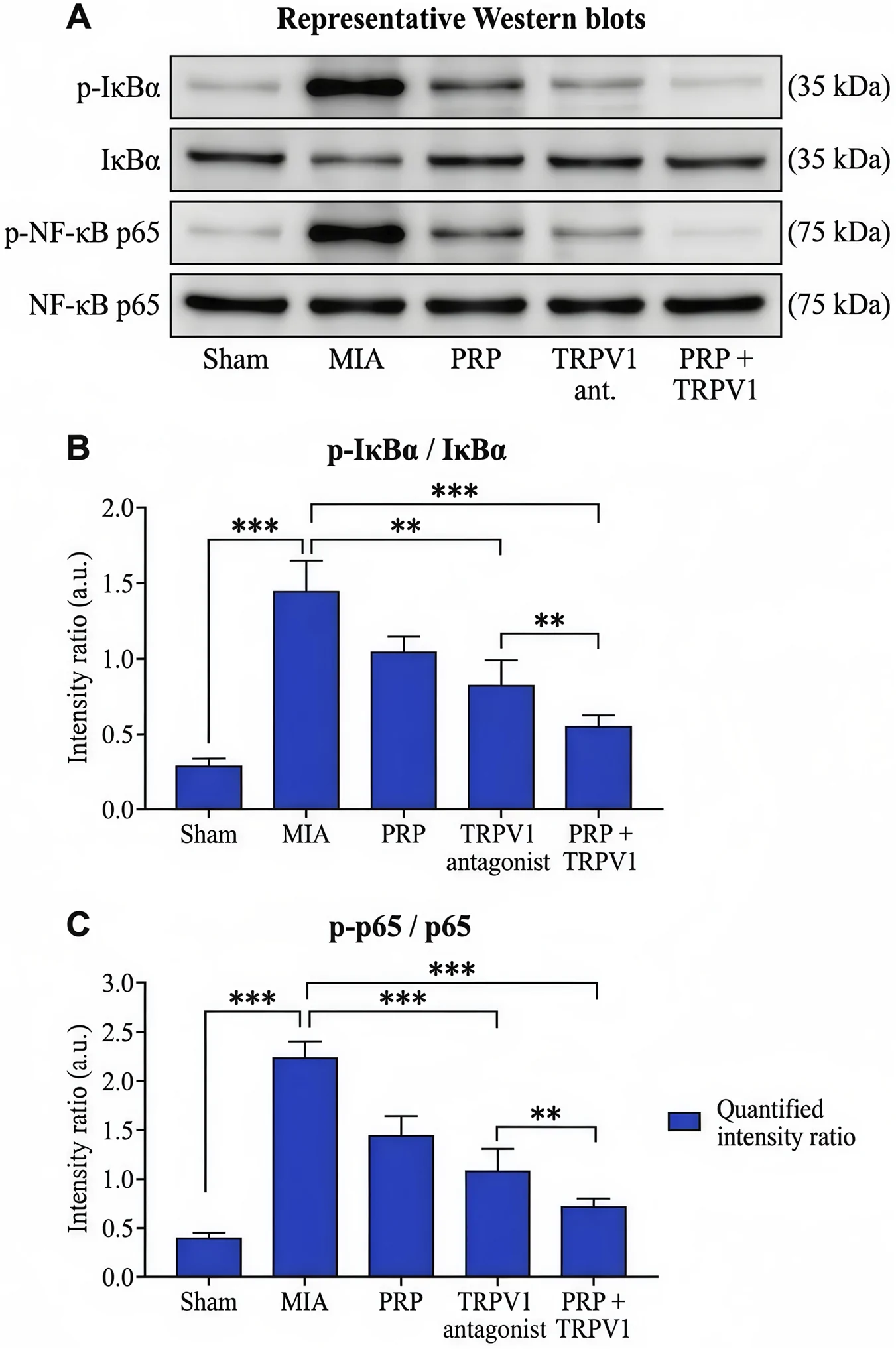
PRP + TRPV1 antagonism suppresses NF-κB pathway activation in synovial tissue. **(A)** Representative Western blots showing p-IκBα, total IκBα, p-NF-κB p65, and total NF-κB p65 in synovial tissue. **(B)** Densitometric ratio of p-IκBα to total IκBα. **(C)** Densitometric ratio of p-p65 to total p65. Data are presented as mean ± SEM (n = 10 per group).

PRP and TRPV1 antagonist monotherapies reduced p-IκBα/IκBα and p-p65/p65 to varying degrees. The combination treatment group showed the strongest suppression of both the indices (Figures 5A–C), supporting broader attenuation of NF-κB-associated inflammatory signaling.

### 3.5 Peripheral sensitization along the joint–DRG axis

Peripheral sensitization markers were assessed at both the joint and DRG levels. After MIA induction, the joint exhibited a sensitizing profile characterized by increased NGF, PGE2, COX-2, and substance P and increased CGRP-positive fiber density. TRPV1, Nav1.8, and CGRP levels were upregulated in ipsilateral DRG, accompanied by increased pERK/ERK and a higher proportion of ATF3-positive neurons, which is consistent with neuronal stress and heightened excitability signatures.

PRP more prominently reduced joint inflammatory and neurotrophic mediators, whereas TRPV1 antagonism more directly attenuated DRG excitability-associated signatures. The combination therapy produced broader attenuation across both the joint and DRG readouts, which is consistent with the concurrent reduction of the levels of peripheral inflammatory drivers and sensory neuron excitability markers. The quantitative summaries are provided in Table 2.

**TABLE 2:** Joint and dorsal root ganglion (DRG) biomarkers of peripheral sensitization after PRP, TRPV1 antagonism, or combination therapy in MIA-induced osteoarthritis model rats.

Tissue	Biomarker	Unit	Sham n=10	MIA n=10	PRP n=10	TRPV1 antagonist n=10	PRP + TRPV1 antagonist n=10	*P*-value (PRP vs. MIA)	*P*-value (TRPV1 antagonist vs. MIA)	*P*-value (PRP + TRPV1 antagonist vs. MIA)	Overall *P*-value (one-way ANOVA)
Joint	NGF	pg/mg protein	18.5 ± 1.1	46.2 ± 2.2	30.1 ± 1.8	32.4 ± 1.9	24.0 ± 1.5	0.002	0.006	<0.001	<0.001
Joint	PGE2	pg/mg protein	96.1 ± 6.3	222.8 ± 11.8	154.3 ± 9.8	166.1 ± 10.5	127.2 ± 8.6	0.004	0.010	<0.001	<0.001
Joint	COX-2 mRNA	fold vs. sham	1.00 ± 0.05	2.82 ± 0.16	1.88 ± 0.13	2.02 ± 0.14	1.44 ± 0.10	0.003	0.008	<0.001	<0.001
Joint	Substance P	pg/mg protein	7.5 ± 0.5	18.5 ± 1.0	12.4 ± 0.8	13.1 ± 0.8	9.9 ± 0.7	0.005	0.012	<0.001	<0.001
Joint	CGRP + nerve fibers	Fibers/mm^2^	11.6 ± 0.9	31.8 ± 2.0	21.6 ± 1.6	20.2 ± 1.5	15.4 ± 1.2	0.007	0.004	<0.001	<0.001
DRG	TRPV1 protein	a.u., normalized	1.00 ± 0.04	1.78 ± 0.08	1.33 ± 0.06	1.19 ± 0.05	1.05 ± 0.04	0.004	<0.001	<0.001	<0.001
DRG	Nav1.8 protein	a.u., normalized	1.00 ± 0.05	1.60 ± 0.07	1.27 ± 0.06	1.29 ± 0.06	1.11 ± 0.05	0.009	0.010	<0.001	<0.001
DRG	CGRP protein	a.u., normalized	1.00 ± 0.04	1.68 ± 0.08	1.31 ± 0.06	1.25 ± 0.05	1.09 ± 0.04	0.006	0.003	<0.001	<0.001
DRG	pERK/ERK	a.u., normalized	1.00 ± 0.05	2.18 ± 0.10	1.52 ± 0.09	1.45 ± 0.08	1.16 ± 0.07	0.002	<0.001	<0.001	<0.001
DRG	ATF3+ neurons	%	5.1 ± 0.7	17.6 ± 1.3	11.2 ± 1.0	10.5 ± 0.9	7.0 ± 0.8	0.004	0.003	<0.001	<0.001

Footnote: data are expressed as the mean ± SEM. *P-*values indicate *post hoc* comparisons *versus* the MIA group. The overall *P-*values are from one-way ANOVA for each biomarker.

### 3.6 Central sensitization in the spinal dorsal horn

**FIGURE 6:**
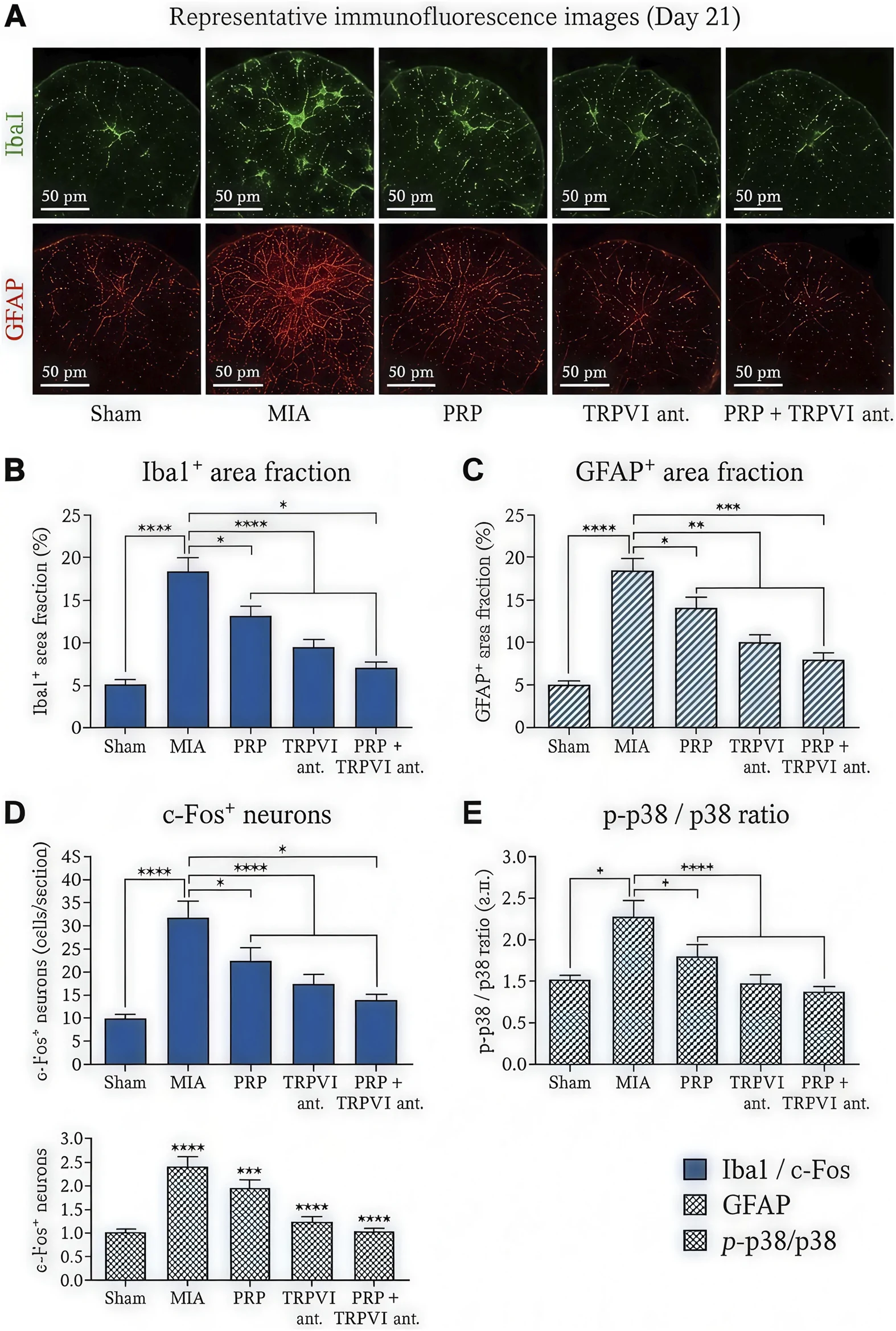
PRP + TRPV1 antagonism reduces dorsal horn glial activation and central sensitization-associated markers in MIA-induced osteoarthritis model rats. **(A)** Representative immunofluorescence images of microglia (Iba1, green) and astrocytes (GFAP, red) in the ipsilateral spinal dorsal horn collected at day 21. Scale bar = 50 μm. **(B)** Quantification of the Iba1-positive area fraction. **(C)** Quantification of the GFAP-positive area fraction. **(D)** Quantification of the c-Fos-positive neurons (cells per section). **(E)** Ratio of p-p38 to total p38 in dorsal horn lysates. Data are presented as the mean ± SEM (n = 10 per group).

Central sensitization-associated readouts were assessed in the spinal dorsal horn. In the MIA group, Iba1 and GFAP levels were elevated, indicating microglial and astrocytic activation. Neuronal activation marker levels were also increased, including higher c-Fos-positive cell counts and an elevated p-p38/p38 ratio, which is consistent with enhanced excitability and inflammatory signaling within spinal pain-processing circuits (Figures 6A–E).

Both monotherapies partially reduced the levels of these central markers. The combination therapy produced the most consistent normalization across readouts, with greater reductions in Iba1 and GFAP levels and shifts in c-Fos and p-p38/p38 toward baseline levels. A consolidated overview is provided in Table 3. In addition, synaptic plasticity-associated proteins (PSD-95, synaptophysin, and NR2B) showed stronger downregulation in the combination group, which is consistent with the attenuation of sensitization-associated synaptic remodeling alongside reduced inflammatory signaling and hyperexcitability.

**TABLE 3:** Spinal dorsal horn neuroglial activation, inflammatory mediators, and synaptic sensitization markers across the experimental groups.

Marker (spinal dorsal horn)	Readout/Unit	Sham n=10	MIA n=10	PRP n=10	TRPV1 antagonist n=10	PRP + TRPV1 antagonist n=10	*P*-value (PRP vs. MIA)	*P*-value (PRP + TRPV1 antagonist vs. MIA)	*P*-value (TRPV1 antagonist vs. MIA)	Overall *P*-value (one-way ANOVA)
Iba1	Iba1+ area fraction (%)	7.1 ± 0.6	18.1 ± 1.1	12.4 ± 0.9	11.7 ± 0.8	8.0 ± 0.7	0.004	0.003	<0.001	<0.001
GFAP	GFAP + area fraction (%)	7.8 ± 0.7	20.7 ± 1.4	14.6 ± 1.1	13.8 ± 1.0	9.3 ± 0.8	0.006	0.004	<0.001	<0.001
c-Fos	c-Fos + neurons (cells/section)	10.2 ± 1.0	31.6 ± 2.1	19.1 ± 1.6	17.9 ± 1.5	11.7 ± 1.2	0.002	<0.001	<0.001	<0.001
p-p38/p38	Intensity ratio (a.u.)	1.00 ± 0.05	2.30 ± 0.11	1.55 ± 0.09	1.46 ± 0.08	1.12 ± 0.07	<0.001	<0.001	<0.001	<0.001
p-ERK/ERK	Intensity ratio (a.u.)	1.00 ± 0.06	2.02 ± 0.12	1.42 ± 0.10	1.36 ± 0.09	1.11 ± 0.08	0.003	0.002	<0.001	<0.001
TNF-α (spinal)	pg/mg protein	10.9 ± 0.8	24.1 ± 1.5	16.8 ± 1.1	16.0 ± 1.1	12.6 ± 0.9	0.004	0.003	<0.001	<0.001
IL-1β (spinal)	pg/mg protein	29.1 ± 2.0	64.9 ± 3.6	45.7 ± 3.2	43.4 ± 3.0	33.2 ± 2.4	0.005	0.003	<0.001	<0.001
PSD-95	Intensity (a.u., normalized)	1.00 ± 0.05	1.69 ± 0.09	1.27 ± 0.07	1.23 ± 0.07	1.07 ± 0.06	0.006	0.004	<0.001	<0.001
Synaptophysin	Intensity (a.u., normalized)	1.00 ± 0.04	1.58 ± 0.08	1.21 ± 0.06	1.20 ± 0.06	1.05 ± 0.05	0.007	0.007	<0.001	<0.001
NR2B	Intensity (a.u., normalized)	1.00 ± 0.05	1.80 ± 0.10	1.36 ± 0.08	1.31 ± 0.08	1.11 ± 0.06	0.004	0.003	<0.001	<0.001

Footnote: data are expressed as the mean ± SEM. *P-*values indicate *post hoc* comparisons *versus* the MIA group. The overall *P-*values are from one-way ANOVA for each marker.

### 3.7 Integrated mechanism-level summary

**FIGURE 7:**
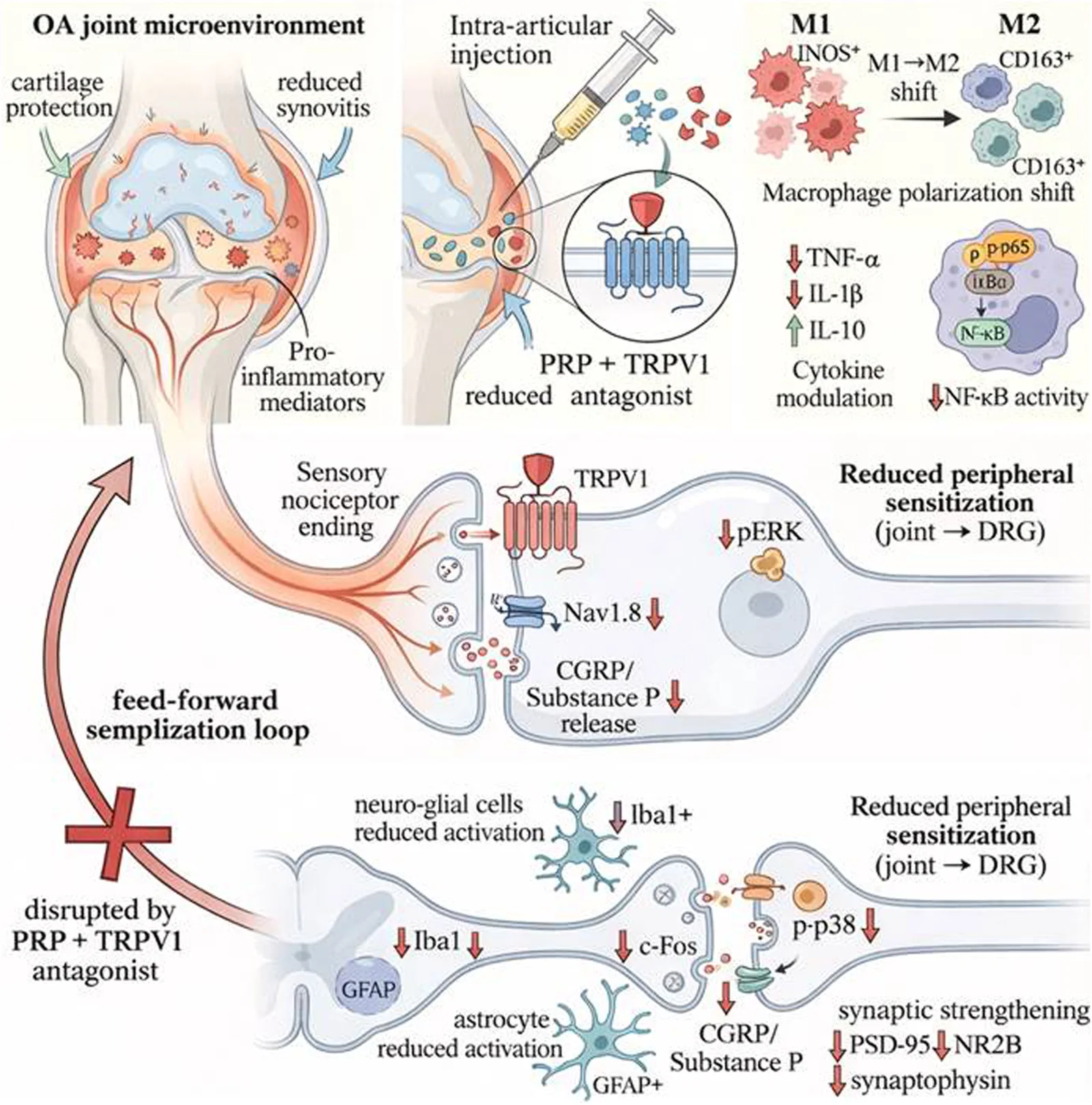
Proposed neuroimmune framework for PRP + TRPV1 antagonism across the peripheral-to-central sensitization axis in osteoarthritis. Schematic illustration of the proposed integrated mechanism. In the joint microenvironment, PRP is proposed to promote a shift away from pro-inflammatory macrophage states, suppress NF-κB-linked inflammatory signaling, and reduce pro-inflammatory mediators (e.g., TNF-α and IL-1β), thereby lowering local sensitizing input to nociceptors. TRPV1 antagonism is proposed to attenuate nociceptive transduction and neuropeptide-associated signaling at sensory terminals and along the joint–DRG axis. Together, the reduced peripheral drive is proposed to coincide with lower spinal dorsal horn glial activation and reduced sensitization-associated synaptic remodeling markers (e.g., PSD-95 and NR2B), which is consistent with the attenuation of central amplification.

Across the behavioral, immune, and neurobiological levels, the data support a coordinated pattern in which PRP is associated with reduced synovial inflammatory load (macrophage polarization, cytokine profile, and NF-κB signaling), whereas TRPV1 antagonism is associated with the attenuation of nociceptor-related sensitization markers. In the combination therapy group, changes were observed concurrently across the synovial immune microenvironment, joint–DRG sensitization indices, and spinal dorsal horn glial/neuronal activation readouts, which is consistent with a more comprehensive attenuation of the sensitization-associated signatures along the peripheral-to-central axis. This integrative framework is summarized in Figure 7.

### 3.8 Clinical translation

Baseline characteristics of the 140 OA patients enrolled between 2023 and 2025 are summarized in Table 4. The cohort primarily comprised middle-aged to older adults with mild-to-moderate pain and functional impairment, with imaging and clinical features reflecting OA heterogeneity.

**TABLE 4:** Baseline demographic, clinical, and imaging characteristics of patients with knee osteoarthritis enrolled between 2023 and 2025 (n = 140).

Domain	Variable	Value n=140
Demographics	Age, years (mean ± SD)	61.8 ± 8.9
	Female, n (%)	92 (65.7)
	BMI, kg/m^2^ (mean ± SD)	26.4 ± 3.5
Disease profile	Symptom duration, years (mean ± SD)	5.8 ± 3.9
	Index knee: right, n (%)	71 (50.7)
	Kellgren–Lawrence grade, n (%)	II: 46 (32.9); III: 63 (45.0); IV: 31 (22.1)
Pain and function	VAS pain (0–10), mean ± SD	6.3 ± 1.4
	WOMAC pain (0–20), mean ± SD	11.7 ± 3.2
	WOMAC stiffness (0–8), mean ± SD	4.7 ± 1.6
	WOMAC function (0–68), mean ± SD	38.5 ± 12.4
	KOOS-QoL (0–100), mean ± SD	44.2 ± 13.6
Alignment and exam	Varus alignment (clinical), n (%)	72 (51.4)
	Knee effusion on exam, n (%)	49 (35.0)
Analgesic exposure	Regular NSAID use in the prior 4 weeks, n (%)	97 (69.3)
	Prior intra-articular injection (steroid or HA), n (%)	29 (20.7)
Comorbidities	Hypertension, n (%)	54 (38.6)
	Type 2 diabetes, n (%)	21 (15.0)
Imaging (MRI/X-ray)	Medial femoral cartilage thickness, mm (mean ± SD)	1.62 ± 0.28
	Medial tibial cartilage thickness, mm (mean ± SD)	1.38 ± 0.25
	Synovitis score (0–3), mean ± SD	2.1 ± 0.9
	Effusion-synovitis present, n (%)	58 (41.4)
	Bone marrow lesion score (0–10), mean ± SD	3.6 ± 2.1
	Medial meniscal extrusion present, n (%)	44 (31.4)

Structural and matrix-related outcomes are summarized in Table 5. Compared with monotherapy, the combination therapy was associated with lower degeneration-related scores, reduced proteoglycan loss, and better preservation of cartilage thickness. In parallel, levels of anabolic markers (COL2A1 and aggrecan) were higher and those of catabolic enzymes (MMP-13 and ADAMTS5) were lower in the combination therapy group, which is consistent with a more anabolic matrix remodeling profile.

**TABLE 5:** Cartilage histopathology and matrix metabolism indicators across experimental groups in MIA-induced osteoarthritis model rats.

Outcome	Sham n=10	MIA n=10	PRP n=10	TRPV1 antagonist n=10	PRP + TRPV1 antagonist n=10	Overall *P*-value
OARSI cartilage score (0–24)	2.2 ± 0.4	13.6 ± 1.0	8.4 ± 0.8	9.1 ± 0.9	5.7 ± 0.7	<0.001
Safranin O loss score (0–6)	0.9 ± 0.2	4.6 ± 0.3	2.9 ± 0.3	3.1 ± 0.3	2.0 ± 0.3	<0.001
Cartilage thickness, μm	193.4 ± 6.8	129.5 ± 6.1	157.4 ± 6.6	151.6 ± 6.9	172.2 ± 6.2	<0.001
COL2A1 IHC-positive area, %	35.1 ± 2.0	17.2 ± 1.5	24.8 ± 1.6	23.5 ± 1.6	29.6 ± 1.7	<0.001
Aggrecan (ACAN) level, a.u. (normalized)	1.07 ± 0.05	0.62 ± 0.05	0.80 ± 0.06	0.78 ± 0.06	0.94 ± 0.05	<0.001
MMP-13 IHC-positive area, %	8.6 ± 0.9	25.3 ± 1.7	16.8 ± 1.4	18.0 ± 1.5	12.6 ± 1.2	<0.001
ADAMTS5 IHC-positive area, %	6.8 ± 0.8	19.2 ± 1.5	13.2 ± 1.2	13.8 ± 1.2	10.2 ± 1.1	<0.001

Footnote: data are expressed as the mean ± SEM. The overall *P-*values are from one-way ANOVA.

Interaction-term analyses are reported in Table 6. In models including the main effects for PRP and TRPV1 antagonism and their interaction, statistically supported interaction effects were observed for pain improvement, cartilage-related outcomes, and sensitization-related measures. Together, the clinical findings align with the animal mechanistic patterns and support a complementary model in which PRP-associated immune microenvironment modulation and TRPV1-associated attenuation of nociceptive sensitization contribute to broader and more consistent therapeutic benefits than with either approach alone.

**TABLE 6:** Interaction-based synergy analyses for pain-, sensitization-, and cartilage-related endpoints under PRP and TRPV1 antagonism.

Endpoint	Expected additive improvement	Observed combo improvement	Synergy (observed − expected)	Interaction term (β)	*P-*value for interaction
Mechanical withdrawal threshold, g	11.8	15.2	3.4	3.1	0.018
Thermal withdrawal latency, s	3.1	4.0	0.9	0.7	0.024
OARSI cartilage score, points (lower is better)	−4.6	−8.1	−3.5	−1.9	0.011
Iba1+ area fraction in dorsal horn, % (lower is better)	−6.8	−10.9	−4.1	−2.6	0.008
DRG TRPV1 protein, a.u. (lower is better)	−0.62	−0.80	−0.18	−0.18	0.015

Footnote: the expected additive improvement was defined as (PRP − MIA) + (TRPV1 − MIA), and the observed combination improvement was defined as (PRP + TRPV1 − MIA). Synergy was computed as (observed − expected). Interaction terms (β) and *P-*values correspond to the PRP × TRPV1 interaction in the factorial models including the main effects for PRP and TRPV1 antagonism and their interaction. Negative values indicate improvement for endpoints, where lower scores represent better outcomes (e.g., OARSI, Iba1 area fraction, and DRG TRPV1).

## 4 Discussion

Our findings demonstrate that PRP combined with TRPV1 antagonism provides superior analgesic outcomes in an MIA-induced OA model along with reduced synovial inflammatory load and improved cartilage matrix-related indices (Glinkowski et al., 2025; Kuang et al., 2023). Notably, the combined regimen was associated with more favorable joint-structure readouts together with coordinated attenuation of synovitis- and degeneration-related markers, while achieving symptomatic pain relief (Ji et al., 2025). As OA pain is increasingly understood to arise from interacting contributions of peripheral inflammation, neuroimmune crosstalk, and central plasticity rather than mechanical wear alone, treatment responses are often heterogeneous and difficult to predict clinically (Abdelaziz et al., 2024). In this context, our data support a neuroimmune framework in which immune-state variability and sensitization-related mechanisms jointly shape divergent pain outcomes across the peripheral, spinal, and behavioral levels (Qiao et al., 2026).

From the perspective of peripheral nociception, TRPV1 is highly expressed in the primary sensory neurons and DRG and is readily sensitized by inflammatory mediators, tissue acidification, and mechanical stimuli, thereby amplifying afferent activity and neurogenic inflammation (Rahman et al., 2024). Consistent with this biology, TRPV1 antagonism was associated with attenuation of DRG excitability-related signatures, supporting its capacity to suppress nociceptive transmission under inflammatory conditions. PRP, in contrast, is positioned upstream within the joint microenvironment. By delivering a repertoire of bioactive molecules (Amodeo et al., 2024; Xu et al., 2025), PRP may modulate the inflammatory crosstalk among synovial cells, immune cells, and chondrocytes; suppress pro-inflammatory programs; and facilitate the restoration of local tissue homeostasis. Conceptually, PRP acts closer to the “generation” of the inflammatory nociceptive drive, whereas TRPV1 antagonism acts closer to the “reception and transmission” of nociceptive signals, providing a complementary rationale for the combined approach (Raut et al., 2025).

At the immune level, the combined regimen was associated with a coordinated shift in synovial macrophage polarization toward anti-inflammatory phenotypes and the suppression of inflammatory amplification-associated signaling (Wu X. et al., 2025). Macrophage polarization is a key component of immune heterogeneity in OA; pro-inflammatory macrophage states sustain synovitis, accelerate catabolic remodeling, and increase the production of pain-promoting mediators. In line with this framework, concurrent decreases in TNF-α/IL-1β alongside the enrichment of IL-10 under combination treatment are consistent with a network-level shift toward resolution. Because NF-κB is a central transcriptional node regulating inflammatory mediator expression and linking immune activation to nociceptor sensitization, the observed suppression of NF-κB-related signaling provides a plausible upstream route through which immune-state remodeling and sensitization-related signatures may be jointly attenuated (Yuan et al., 2024).

In this clinical cohort, baseline imaging and phenotypic profiles highlighted the diversity of OA pathology, including variable degrees of cartilage degeneration and synovitis. Such heterogeneity provides an appropriate premise for mechanism-informed interpretation of treatment response. As reported previously, PRP can improve pain and function in specific patient subgroups, but the outcomes vary by inflammatory state, disease stage, and procedural factors (Yin et al., 2024). By adding TRPV1 antagonism as a neuro-targeted component, the present study addresses an additional dimension of response variability that is plausibly driven by sensitization-related mechanisms. This combined strategy may be particularly relevant for subgroups characterized by stronger sensitization signatures or inflammation-amplified nociceptive gain.

A central mechanistic question raised by our findings is how immune remodeling relates to reduced central sensitization. One parsimonious pathway is that TRPV1 blockade reduces peripheral nociceptive input, thereby secondarily decreasing dorsal horn glial activation and neuronal hyperexcitability. A second, non-mutually exclusive pathway is that the altered cytokine milieu (e.g., increased IL-10 with reduced TNF-α/IL-1β) may more directly modulate DRG excitability and neuroimmune coupling, thus reducing the strength of the afferent drive that maintains central amplification. Importantly, the present study provides coordinated, cross-level evidence that is consistent with both pathways, but it does not definitively assign causal weight to either route. Discriminating these mechanisms will require targeted experiments, such as DRG electrophysiology under cytokine perturbation, local neutralization of IL-10, or factorial designs that separate peripheral input blockade from immune-state shifts.

These results also align with a broader view that recovery from sensitization reflects distributed plasticity across pain-processing circuits rather than a single-node effect. Evidence from other domains of neural adaptation and recovery indicates that neural signatures can reorganize in response to injury and external inputs and that environmental and behavioral influences may shape the stability and direction of recovery trajectories (Liu et al., 2025; Pirdehghan et al., 2021; Fan et al., 2023; Fan et al., 2026). In this comparative context, the reductions in dorsal horn glial activation and synaptic sensitization-associated readouts observed after combination therapy are consistent with a “plasticity-restoring” directionality, in which diminishing peripheral inflammatory drive and nociceptive transmission support rebalancing of the central pain-processing states.

More broadly, symptom heterogeneity and inter-individual variability have been linked to systems-level neural signatures, including brain asymmetry patterns and cortical thickness correlates of comorbidity and symptom severity (Correction: Brain asymmetry and cortical, 2025; Cortical thickness alterations serve as, 2023; Neural structural adaptation and symptom, 2023). Although our study focuses on spinal sensitization, situating dorsal horn plasticity within this broader neurobiological literature reinforces the interpretation that sensitization phenotypes reflect distributed adaptations that vary across individuals and may, therefore, shape analgesic responsiveness.

Several limitations of this study should be acknowledged. The MIA model produces rapid pain and inflammatory changes that are well suited for mechanistic analysis, but it does not fully recapitulate the slower, chronic degenerative course of human OA. Our animal experiments ended at day 21 and, therefore, primarily capture early-to-intermediate sensitization and immune remodeling. The durability of macrophage reprogramming and cytokine resolution is not established, and macrophage phenotypes may revert toward pro-inflammatory states after treatment cessation. Longer follow-up with repeated synovial immunophenotyping, extended behavioral testing, and longitudinal spinal readouts will be important to evaluate the durability and potential rebound of the immune and sensitization signatures. In addition, TRPV1-based interventions can exhibit tissue- and cell-specific effects; thus, dosing, delivery route, and safety profiling require careful optimization for clinical translation. Future studies should refine dose and intervention frequency, extend follow-up duration, and test stratification strategies that incorporate inflammatory and sensitization phenotypes to identify patients that are most likely to benefit from this strategy.

## 5 Conclusion

In summary, combining PRP with TRPV1 antagonism was associated with stronger and more stable analgesic effects than either of the monotherapies in an MIA-induced OA model, accompanied by a coordinated shift toward an anti-inflammatory synovial milieu, suppression of NF-κB-linked inflammatory activation, attenuation of peripheral sensitization markers along the joint–DRG axis, and reduced spinal dorsal horn glial/neuronal activation signatures. In a translational clinical cohort, interaction-term analyses further supported benefits beyond simple additivity across pain-, sensitization-, and cartilage-related outcomes. Together, these findings support a mechanism-oriented view that addressing both immune microenvironment heterogeneity and sensitization-related processes may yield more consistent therapeutic responses in OA, and they motivate future studies with longer follow-up and stratified designs to define durability, safety, and patient subgroups that are most likely to benefit from this combined strategy.

## Data Availability

The colorectal cancer RNA-sequencing, DNA methylation, and clinical data were obtained from the National Cancer Institute Genomic Data Commons. The datasets were derived from the TCGA Colon Adenocarcinoma project, with the project identifier TCGA-COAD, available at https://portal.gdc.cancer.gov/projects/TCGA-COAD, and the TCGA Rectum Adenocarcinoma project, with the project identifier TCGA-READ, available at https://portal.gdc.cancer.gov/projects/TCGA-READ. The relevant RNA-sequencing, DNA methylation, and clinical information can be accessed through these project pages. The normal tissue gene-expression data were obtained from the Genotype-Tissue Expression project, specifically the GTEx Analysis V8 release. The corresponding dbGaP accession number is phs000424.v8.p2. The normal colon tissues included Colon–Sigmoid and Colon–Transverse samples. The GTEx dataset page is available at https://gtexportal.org/home/datasets, and the adult GTEx data download page is available at https://www.gtexportal.org/home/downloads/adult-gtex. The eQTL summary statistics were also obtained from the GTEx Analysis V8 release under dbGaP accession number phs000424.v8.p2, with Colon–Sigmoid and Colon–Transverse tissues included in the analysis. The relevant QTL datasets can be accessed through the GTEx QTL download page at https://www.gtexportal.org/home/downloads/adult-gtex#qtl. The colorectal cancer GWAS summary statistics were obtained from the IEU OpenGWAS database, dataset ID ebi-a-GCST90018808 (GWAS Catalog accession GCST90018808).
